# Scientometric Analysis of Hiking Tourism and Its Relevance for Wellbeing and Knowledge Management

**DOI:** 10.3390/ijerph19148534

**Published:** 2022-07-13

**Authors:** Ángel Acevedo-Duque, Gonzalo R. Llanos-Herrera, Elizabeth Emperatriz García-Salirrosas, Selene Simón-Isidoro, Agustín Pablo Álvarez-Herranz, Rina Álvarez-Becerra, Lisette C. Sánchez Díaz

**Affiliations:** 1 Public Policy Observatory, Universidad Autónoma de Chile, Providencia 7500912, Chile; 2 School of Economics and Business, Universidad Finis Terrae, Santiago 7501015, Chile; gllanos@uft.cl; 3 Faculty of Management Science, Universidad Autónoma del Perú, Lima 15842, Peru; egarciasa@autonoma.edu.pe; 4 Programa de Doctorado en Economía y Empresa, Universidad Castilla la Mancha, 16071 Cuenca, Spain; mselene.simon@alu.uclm.es; 5 Facultad de Ciencias Sociales, Universidad Castilla la Mancha, 16071 Cuenca, Spain; agustin.alvarez@uclm.es; 6 Graduate School, Universidad Nacional Jorge Basadre Grohmann, Tacna 23001, Peru; ralvarezb@unjbg.edu.pe; 7 Dirección Departamento de Auditoría, Contabilidad y Control de Gestión, Universidad Católica del Norte, Antofagasta 1270375, Chile; lisette.sanchez@ucn.cl

**Keywords:** tourism, hiking, trekking, trail, trail tourism

## Abstract

Hiking is a sports activity that takes place in the natural environment. From the point of view of well-being, it is an aerobic activity that prevents and improves cardiovascular diseases. According to data provided by the United Nations, within the framework of the International Year of Mountains, mountain tourism represents around 15% to 20% of total world tourism revenue. This approach aims to critically analyze the scientific production on trail tourism (HT) with contributions from authors from around the world from 1991 to 2022, in order to respond to the connection between this research, knowledge management and the sustainable development of the industry. Key knowledge contributions are examined using a scientometric approach as a method (spatial, production, impact, and relational) based on registry data stored in the Web of Science (JCR and ESCI). Regarding the results, there has been an increase in scientific production in the last decade, which is manifested in the quality of the publications.

## 1. Introduction

The world is currently facing an unprecedented global health crisis; COVID-19 is spreading human suffering, destabilizing the global economy, and dramatically changing the lives of billions of people around the world [1]. The contribution of tourism to economic growth and development can also have a collateral effect on health and well-being, bringing conscious action to Sustainable Development Goal 3 (SDG3), this type of activity also focuses, directly or indirectly, on other goals across the board, SDG8, SDG9, SDG12 and SDG13 to name a few [2]. Specifically, it has been included in some of the goals of goals 8, 12, 14 and 15, related, respectively, to inclusive and sustainable economic growth, consumption and production and the sustainable use of land and ocean ecosystems and marine resources. [3].

The latter being focused from the perspective of trail tourism and how to enjoy majestic landscapes, undisturbed forests, abundant biodiversity and natural heritage sites [4]. It is often one of the main reasons for tourists to visit a destination in a pandemic crisis [5]. Trail tourism can play an important role not only in the conservation and preservation of biodiversity, but also in the respect of terrestrial ecosystems, due to its efforts to reduce waste and consumption, conservation of native flora and fauna and awareness-raising activities [6] in this broad sense, and on the basis of which it can generate interest in new physical, recreational and sporting activities [7]. Considering the latter, it could be said that it is one of the most chosen activities by those people who wish to increase and improve their quality of life.

The interest of this research is focused on analyzing the studies carried out on hiking or walking tourism [8]. This trend is observed worldwide, as there has been a considerable exponential increase in recent years in the studies carried out by researchers worldwide [9]. This type of activity stands out from the rest because a large number of people are looking to hiking as an alternative to improve their quality of life, to get to know new places or simply use it as a complement to their usual activity, demanding quality and diversity in the physical sports activities carried out in the natural environment, as well as learning and managing new knowledge [10]. 

Hiking offers multiple possibilities for development as one of the most efficient forms of sustainable tourism [11]. Well-planned trekking tourism can be a constant source, not only of quality of life for those who practice it, but also of ample economic income and consequently an opportunity for the development of local, primarily rural populations [12]. Through this type of tourism it is also possible to obtain resources and mechanisms for environmental protection, in order to extend the natural conservation of a particular area. Although it is important to ensure optimal implementation, as there is also the risk of devastating the environment and destroying the natural capital in order to attract visitors [13].

It is not usually a question of having one type or the other, but of seeking a balance between the two, in order to generate sufficient income to turn the project into something profitable, but with sufficient care to ensure that the natural values are not depleted or diluted [14]. It is in this sense that alternatives such as trekking are becoming increasingly popular, as they involve many of the values of health tourism, artisanal tourism and information management based on knowledge, which is what is recommended to prioritize with long-term sustainable development in mind [15].

Therefore, the aim of this article is to critically analyze the scientific production on hiking and trekking as a source of tourism knowledge that has had contributions from affiliated authors in several countries taking into account the global references on this field that are increasingly identified and highlighted in order to manage pre-existing knowledge and promote a connection between research and the improvement of the current understanding of this sustainable industry [16]. Thus, based on articles published in Web of Science (WOS) journals, this paper conducted a scientometric analysis of knowledge production in walking and hiking in tourism [17] to identify the thematic areas where the issue of sustainability in tourism is currently being debated. Interest in trail tourism has grown persistently over the last three decades. This research seeks to provide an answer regarding the relationship between walking and hiking tourism research and its contribution to society at large.

## 2. Background

### 2.1. Hiking as Sustainable Development for Tourism

Hiking is synonymous with rambling, i.e., walking mainly along paths and trails [17]. It is a mixture of sport and tourism activities, mainly taking place in natural environments. Hiking has not only become, in many cases, one of the main routes for sustainable tourism with a focus on health and wellbeing in rural communities but can even become the backbone of a complete leisure network that also includes and connects the many attractions a region has to offer [18]. Job and wealth creation is one of the most important challenges facing not only small municipalities today, but society as a whole. However, in the case of municipalities with few inhabitants, the problem is aggravated by the fact that the progressive reduction in jobs can lead to their disappearance [19]. Large cities (which, despite administrative divisions, are sometimes spread over several municipalities), in their eagerness to develop at all costs, have embarked on strategic plans based essentially on competitiveness in this type of crazy tourism.

For tourism, 2021 and, so far 2022, have been years of learning and adaptation [20]. It has been shown that only through collaboration can the sector overcome challenges and seize new opportunities [8]. The UNWTO, by bringing together the will of the tourism community and offering concrete measures, has guided the tourism response with the aim of not only reviving the sector, but also making it increasingly inclusive, innovative and sustainable [21]. For this organization, mountain or trekking tourism is a type of tourism activity that takes place in a defined and delimited geographical space such as hills or mountains, with characteristics and attributes inherent to a given landscape, topography, climate, biodiversity (flora and fauna) and the local community, encompassing a wide range of outdoor leisure and sport activities [22].

**Figure 1:**
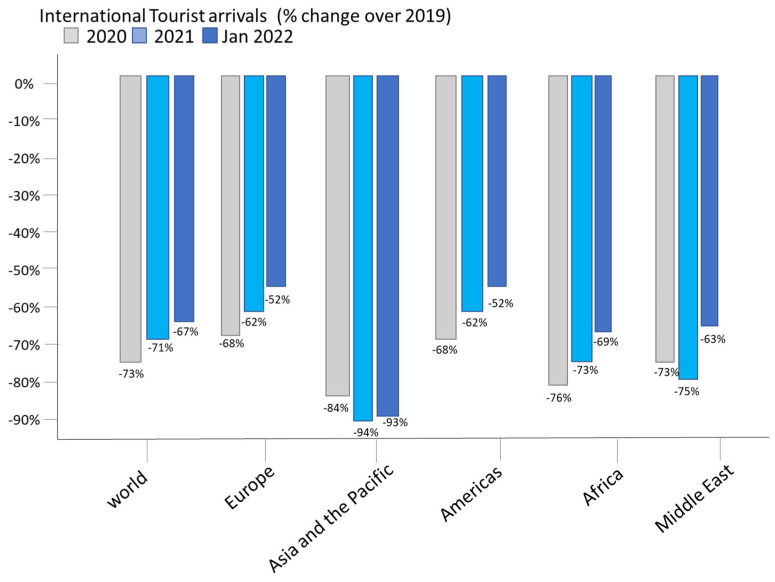
Source: World Tourism Organization (UNWTO). Change over 2019 (provisional data). Data collected by UNWTO, March 2022. Published: 25 March 2022.

All regions experienced a significant rebound in January 2022, although it should be noted that this compares with low levels recorded in early 2021 [23], Europe (+199%) and the Americas (+97%) continue to show the best results, with international arrivals still at half of pre-pandemic levels. The Middle East (+89%) and Africa (+51%) also recorded an increase in January 2022 compared with 2021 [24], but these regions experienced a decline of 63% and 69%, respectively, compared with 2019. Although Asia-Pacific saw a 44% year-on-year increase, several destinations remained closed to non-core travel, resulting in the largest decline in international arrivals since 2019 (−93%) (See Figure 1).

By subregion, the best performance has been in Western Europe, with four times the number of arrivals recorded in January 2022 compared with 2021 data, but 58% fewer than in 2019. Both the Caribbean (−38%) and Southern Mediterranean Europe (−41%) have shown the fastest rates of recovery compared with 2019 levels [25]. In fact, several Caribbean islands, as well as the Asia-Pacific, along with some small European and Central American destinations have the best performance compared with 2019: Seychelles (−27%), Bulgaria and Curaçao (both −20%), El Salvador (−19%), Serbia and Maldives (both −13%), Dominican Republic (−11%), Albania (−7%) and Andorra (−3%). Bosnia and Herzegovina (+2%) even surpassed pre-pandemic levels. Among the top destinations, Turkey and Mexico experienced declines of 16% and 24%, respectively, compared with 2019 [25]. 

UNWTO brings together political leaders from across the globe for a strong and coordinated response [26], governments, destinations, other UN agencies and international organizations have met at key international events and joined forces to rethink tourism. Institutional coordination has proven to be crucial in finding solutions that contribute to smarter, greener and safer tourism. Walking tourism has great potential to stimulate local economic growth and social change due to its complementarity with other economic activities, its contribution to GDP and job creation, and its capacity to promote the dispersion of demand over time (combating seasonality) and across the territory [27].

### 2.2. Benefits of Knowledge Management in Hiking Tourism

Hiking is gaining importance since it is practiced as a healthy, wellness, leisure, recreational and educational activity carried out both on the seacoast and in the mountains [28]. It focuses on the tourist as an active element, seeking a real identity in relation to a territory or a place, while travelling through natural and rural environments or in the field of sport. Hiking acts by promoting the development of a local identity focused on the cultural resources of a given territory [29,30]. It stands out because many times there are paths that represent the main communication routes of other times or places and also because of the importance of the great cultural centers destined for religious or pilgrimage tourism.

It is interesting to mention that this type of tourism seeks to bring people closer to the natural environment and to the knowledge of an area through heritage and traditional ethnographic and cultural elements, using especially dirt tracks, old bridle paths and roads, ravines and royal roads, forest roads and others. Significantly impacting on economic, environmental, cultural and social sectors (Table 1).

It is evident that there is an intrinsic and strong relationship between hiking and related factors in the benefits that hiking can bring, especially in terms of the well-being of people and the economic factors to keep them sustainable [46]. This activity manages other important benefits such as promoting heritage, culture, architectural landscapes of the traditional rural environment and the development of new opportunities beyond agricultural production, based on a main resource: the landscape [47]. This resource is understood as having natural and cultural value and must be managed under the paradigm of sustainable development [48]. In addition to interrelating hiking with other forms of tourism, it generates direct benefits to local populations, and therefore, to the tourist destination.

## 3. Materials and Methods

Many researchers around the world have studied how publications generate changes in the world of knowledge and how these in one way or another impact on the strategies and decisions of nations [49,50], and the impact of new research article writing technologies [50,51]. Major research topics include impact measurement, article reference sets for investigating the impact of journals and institutes, understanding scientific citations, mapping scientific fields, and producing indicators for use in policy and management contexts. In practice, there is significant overlap between scientometrics and other scientific fields such as bibliometrics, information systems, information science and science policy.

The above represents some of the ways in which scientometrics has gained popularity in recent decades based on the documented knowledge available and shows interest in quantitative analysis of scientific output (especially scientific articles) to investigate the development, structure, dynamics, trends and relationships of scientific practice, and especially, the exponential growth of the variable under study to obtain quantitative results [52] and to determine the growth of knowledge within various fields. The development of this research complements the exposed literature review with a scientometric and bibliometric analysis inspired mainly by the methodology employed in 2018 by researchers Llanos-Herrera and Merigo [53]. This methodology analyses publications, citations and sources [54]. One of the most relevant benefits is the systematization of the information, and through which, information at the level of articles, academic journals, authors, universities and countries can be considered [55]. This research technique has been consolidated over time as an effective way to obtain an overview of the research conducted on different topics of interest, particular journals and even the study of articles that cite a specific article, such as a seminal article. This methodology has evolved and improved over the years in its understanding and importance [56].

In terms of methodology, we analyze the evolution and development of scientific knowledge from an economic and social approach, and in academic research, one of the most relevant aspects to take care of is the impartiality of the data that are the objects of analysis [57,58,59,60]. For this reason, the analysis below is based on data obtained from the WoS platform provided by Clarivate Analytics, which contains more than 171 million articles with around 1.9 billion citations. Although there are other databases, such as SciELO (Scientific Electronic Library Online) and SCOPUS, this study is based on WoS in order to standardize the information obtained by following only one standardized source [61,62].

Thus, based on spatial scientometrics [63,64], to start with the development of this research, the researchers agreed to use the cross-reference of any of these three terms: “hiking”, “trekking” and “trail”, with “tourism”. A review of the results of this search was then carried out, obtaining through this search a highly effective search in terms of the subject of trail tourism. At the end of this review, we discarded those articles that fortuitously fulfilled the search criteria but were not clearly related to trail tourism.

As a first step, the legitimacy of conducting the research was determined based on the observation of a collection of articles that manages to maintain a critical mass of production between the generation of new knowledge and its obsolescence [65,66]. The procedure was carried out in February and March 2022, obtaining a total of 820 articles distributed in the following types of publications: articles (757), review articles (26), early access (22) and proceedings articles (15). In order to maintain a good quality standard, this research concentrated on articles and review articles, of which, after a one-to-one review, an analysis base of 781 articles was obtained. Through the VOSviewer software [67], taking into account both the thematic difference expressed by a set of “keywords plus” that can be reduced to the main “keywords plus” [68], according to Zipf’s law [68] and the existence of potential concentrations in terms of prolific journals and authors.

Bradford’s law was employed to estimate a possible fit of journal concentration zones [69,70]. In order to identify a core concentration of journals where authors engage in deep path tourism discussion, Lotka’s law [71,72] was used. To estimate the concentration of authors and distinguish the set of prolific authors [73,74], following a meta-analytic scientometric approach [75] of production, impact and relatedness that relationally focused on establishing levels of co-authorship between authors with their affiliations and their collaborators, using VOSviewer and Pajek [76,77].

The information provided by WoS was first analyzed in a general way, mainly based on the abstracts, with the intention of understanding the structure of trail tourism research. The data was also reviewed in terms of the number of articles and citations [78]. The analysis then focused on journals, authors, institutions and countries. For this purpose, the number of articles, number of citations and different quality indices were considered. Next, an analysis was carried out under the perspective of two-dimensional maps to understand the structure and how the research in trail tourism had been designed in terms of linkage between authors, citations between authors, use of keywords and journals.

## 4. Results

This section presents the results of the data analyses associated with the citation structure, the H-index and the most influential articles, authors, institutions and countries with respect to trail tourism management, carrying out the purpose of the present research.

### 4.1. Results of the Number of Trail Tourism Publications from 1991 to 2022 in WoS

**Figure 2:**
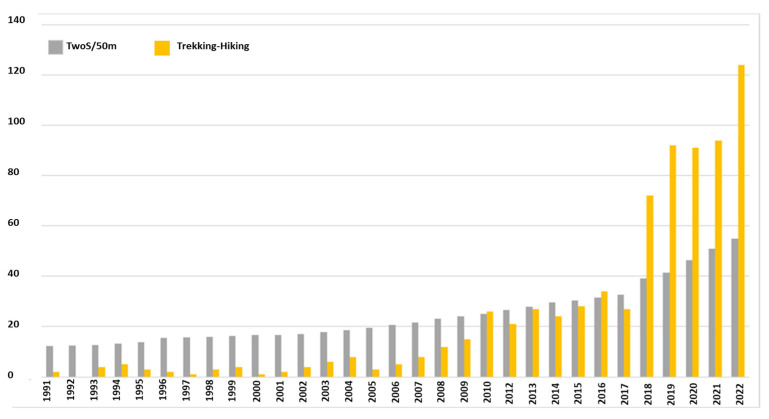
Number of trail tourism publications from 1991 to 2022 in WoS.

It is important to note that NPHT is the total number of publications on hiking and NTP is the total number of articles published in WoS. The yellow bars represent the number of articles published on walking and the grey bars indicate the ratio NTP/50,000 (Figure 2).

In this way, this article explores areas of interest in hiking and trekking as tourism and scientific articles that address this discipline, taking into account all its contexts, in particular sustainability, in itself has been part of the tradition of nations since time immemorial, in fact it is relatively recent its consolidation as a tourist alternative, which is also an important way of relief from the urban saturation that prevails in these times. This is reflected, in a broad sense, in the growing attention that this sub-discipline has received from discourses, studies and works of high impact carried out during the last few years, both in journals dedicated exclusively to this activity and in other scientific areas that are analyzed, not only has it become in many cases one of the main ways for the sustainable tourism of rural communities, but it can even constitute by itself, in the axis of a complete leisure network, where the multiple attractions that a region can offer are also included and connected.

### 4.2. Results of the Evolution of the Citation Structure

As a first step in this process, the general structure of the research was reviewed. Thus, Figure 1 shows the evolution of publications in WoS (articles and reviews) that consider hiking and tourism, contrasted with an indicator of the evolution of general publications in WoS. The increase of interest in trail tourism is remarkable. Over the last 20 years, the annual growth of articles considering hiking in the framework of tourism has increased at an average annual rate of 32% (compared with the annual growth of total WoS publications of 6%), which indicates that the growth in interest in this topic is well above the average growth of WoS publications. Although the growth of research in this area has been slowing down, significant growth rates have been observed in recent years.

Table 2 shows the ranking of the 35 most cited journals in the last decade that include trails and tourism in their topics. It can be seen that the *Journal of Environmental Management* concentrates the highest number of citations in this period and stands out dramatically from the rest when considering the total number of citations where trail tourism is incorporated. When taking the perspective of total citations in articles on trail tourism, it contributes more than 13% of the total citations of all journals in this topic. It also excels in the average number of citations in this topic (leaving out the journals with only one publication in this analysis), reaching a total of 63 (more than 12 average citations per article than its closest follower, *Monitoring and Assessment*).

### 4.3. Results of the H-Index Applied to Hiking Research

The H-index is a key element to understand the importance of a group of articles. It is the number of articles “x” that have at least “x” citations. Thus, if an author has seven articles with 15, 14, 12, 7, 5, 3 and 0 citations, respectively, his H-index would be 5, since he has at least five articles with 5 or more citations. In particular, the H-index for trail tourism is 51, with at least 51 articles with 51 or more citations.

The publications considering hiking belong to 390 journals. Table 3 shows a ranking of the 20 most influential journals in this field, using as an indicator in the first instance the H-index (of four or more) and in the second instance the number of citations (118 or more), both for hiking. Thus, according to the H-index for hiking, the most influential journals are the *Journal of Environmental Management*, *Tourism Management* and the *Journal of Sustainable Tourism*. Among these journals, more than 24% of the citations in hiking are concentrated.

### 4.4. Results of the Most Influential Articles in Trekking

One of the essential aspects to analyze in academic publications is the ranking of articles according to the total number of citations they have received. Although many variables can be considered to evaluate the influence of an article in the academic environment, the number of citations is a simple and objective measure of the interest and influence of a hiking article in the scientific community. Table 4 presents the 30 most cited articles of all time in research involving hiking.

**Table 4:** The 30 most cited papers regarding hiking and trekking.

R	Source Title	TC	Article Title	Authors	Year	C/Y
1	FEE	121	Recent advances in recreation ecology and the implications of different relationships between recreation use and ecological impacts	Monz, CA; Pickering, CM; Hadwen, WL	2013	13.4
2	EMA	100	A study on the determination of the natural park’s sustainable tourism potential	Cetin, M; Zeren, I; Sevik, H; Cakir, C; Akpinar, H	2018	25.0
3	PO	89	Spatial and temporal dynamics and value of nature-based recreation, estimated via social media	Sonter, LJ; Watson, KB; Wood, SA; Ricketts, TH	2016	14.8
4	EMA	82	Evaluating the recreation potential of Ilgaz Mountain National Park in Turkey	Cetin, M; Sevik, H	2016	13.7
5	JTTM	71	Nature-based tourism: motivation and subjective well-being	Kim, H; Lee, S; Uysal, M; Kim, J; Ahn, K	2015	10.1
6	TM	70	Visitor monitoring along roads and hiking trails: How to determine usage levels in tourist sites	Wolf, ID; Hagenloh, G; Croft, DB	2012	7.0
7	LUP	70	Aesthetic appreciation of the cultural landscape through social media: An analysis of revealed preference in the Dutch river landscape	Tieskens, KF; Van Zanten, BT; Schulp, CJE; Verburg, PH	2018	17.5
8	TG	64	Nature-based tourism and neoliberalism: Concealing contradictions	Duffy, R	2015	9.1
9	JEM	60	The impacts of trail infrastructure on vegetation and soils: Current literature and future directions	Ballantyne, M; Pickering, CM	2015	8.6
10	JEM	60	Impacts of informal trails on vegetation and soils in the highest protected area in the Southern Hemisphere	Barros, A; Gonnet, J; Pickering, C	2013	6.7
11	JST	56	Developing sustainable tourism through adaptive resource management: a case study of Machu Picchu, Peru	Larson, LR; Poudyal, NC	2012	5.6
12	CIT	52	Geosite assessments: comparison of methods and results	Strba, L; Rybar, P; Balaz, B; Molokac, M; Hvizdak, L; Krsak, B; Lukac, M; Muchova, L; Tometzova, D; Ferencikova, J	2015	7.4
13	TM	51	Residents’ perceptions of wine tourism development	Xu, SY; Barbieri, C; Anderson, D; Leung, YF; Rozier-Rich, S	2016	8.5
14	EJRS	51	Recognition of surface flow processes influenced by roads and trails in mountain areas using high-resolution topography	Tarolli, P; Calligaro, S; Cazorzi, F; Dalla Fontana, G	2013	5.7
15	AT	50	Concentration of fecal cortisol metabolites in chamois in relation to tourist pressure in Tatra National Park (South Poland)	Zwijacz-Kozica, T; Selva, N; Barja, I; Silvan, G; Martinez-Fernandez, L; Illera, JC; Jodlowski, M	2013	5.6
16	LUP	49	Recreational trails are an important cause of fragmentation in endangered urban forests: A case-study from Australia	Ballantyne, M; Gudes, O; Pickering, CM	2014	6.1
17	LS	49	Exploring relationships between recreation specialization, restorative environments and mountain hikers’ flow experience	Woran, B; Arnberger, A	2012	4.9
18	BC	48	Balancing the benefits of ecotourism and development: The effects of visitor trail-use on mammals in a protected area in rapidly developing China	Zhou, YB; Buesching, CD; Newman, C; Kaneko, Y; Xie, ZQ; Macdonald, DW	2013	5.3
19	AG	48	Digital footprints: Incorporating crowdsourced geographic information for protected area management	Walden-Schreiner, C; Leung, YF; Tateosian, L	2018	12.0
20	JAB	47	Outdoor recreation causes effective habitat reduction in capercaillie Tetrao urogallus: A major threat for geographically restricted populations	Coppes, J; Ehrlacher, J; Thiel, D; Suchant, R; Braunisch, V	2017	9.4
21	AAAG	47	Making a living the Hmong way: An actor-oriented livelihoods approach to everyday politics and resistance in Upland Vietnam	Turner, S	2012	4.7
22	ATR	45	The Inca Trail experience: Does the journey matter?	Cutler, SQ; Carmichael, B; Doherty, S	2014	5.6
23	TG	44	Research frontiers glacier tourism: A scoping review	Welling, JT; Arnason, T; Olafsdottir, R	2015	6.3
24	JORTRPM	43	Current knowledge and future research directions for the monitoring and management of visitors in recreational and protected areas	Pickering, C; Rossi, SD; Hernando, A; Barros, A	2018	10.8
25	LUP	43	A portfolio of natural places: Using a participatory GIS tool to compare the appreciation and use of green spaces inside and outside urban areas by urban residents	Bijker, RA; Sijtsma, FJ	2017	8.6
26	A	42	Is tourism damaging ecosystems in the Andes? Current knowledge and an agenda for future research	Barros, A; Monz, C; Pickering, C	2015	6.0
27	JEM	41	The effect of minimum impact education on visitor spatial behavior in parks and protected areas: An experimental investigation using GPS-based tracking	Kidd, AM; Monz, C; D’Antonio, A; Manning, RE; Reigner, N; Goonan, KA; Jacobi, C	2015	5.9
28	S	40	Estimating the economic impacts of a small-scale sport tourism event: The case of the Italo-Swiss Mountain Trail CollonTrek	Duglio, S; Beltramo, R	2017	8.0
29	MRD	39	Non-native plant invasion in relation to tourism use of Aconcagua Park, Argentina, the highest protected area in the Southern Hemisphere	Barros, A; Pickering, CM	2014	4.9
30	JST	38	Ecotourism as a conservation tool and its adoption by private protected areas in Brazil	Pegas, FD; Castley, JG	2014	4.8

It is important to note the Abbreviations: A, Ambio; AAAG, Annals of the Association of American Geographers; AG, Applied Geography; AT, Acta Theriologica; ATR, Annals of Tourism Research; BC, Biological Conservation; CIT, Current Issues in Tourism; EJRS, European Journal of Remote Sensing; EMA, Environmental Monitoring and Assessment; FEE, Frontiers in Ecology and the Environment; JAB, Journal of Avian Biology; JEM, Journal of Environmental Management; JORTRPM, Journal of Outdoor Recreation and Tourism-Research Planning and Management; JST, Journal of Sustainable Tourism; JTTM, Journal of Travel & Tourism Marketing; LS, Leisure Sciences; LUP, Landscape and Urban Planning; MRD, Mountain Research and Development; PO, Plos One; S, Sustainability; TG, Tourism Geographies; TM, Tourism Management.

From the information in Table 4, the articles by [79,80], which exceed 100 citations, stand out for their number of citations, with the former focusing on the impacts of ecological recreation and the latter on the potential of sustainable tourism. The article by [81] also stands out for its high number of citations per year, second only to the article by [81].

Table 4 shows, significantly from the data exploration of this research work, the 30 most cited articles on hiking and trekking. Showing the interest of the most influential authors in this discipline of knowledge, their works point to different lines of research that are immersed in the gaps that exist to continue contributing to sustainable tourism. In this way the exposed authors highlight in their research the increasing demand of consumers to “experience” a destination in an authentic way, as well as the growing popularity of active tourism, making walking tourism more and more important beyond trekking activities, as it shows a destination in its entirety, including local nature and culture.

### 4.5. Results of an Overview of the Most Productive and Influential Authors

As can be seen in Figure 1, research incorporating hiking began to appear in WoS records in 1991, alluding to tourism issues based on sustainable development and crisis analysis [82,83]. An important aspect in the review of hiking research is to recognize which are the authors with the greatest presence and influence on this subject. To this end, Table 5 presents a list of the 12 authors with the highest number of articles on hiking. It is important to mention that the number of articles is a referential measure, since other potentially relevant aspects such as the length of the article, the quality of the journal that publishes it, the number of authors, etc., are not considered under this analysis.

As shown in Table 5, Pickering, from Australia, is the most influential author with more than 1191 citations, followed by Leung from the USA, with H-indexes of 22 and 10, respectively. Between them, these two authors account for more than 16% of the total citations in hiking research.

### 4.6. Results the Most Productive and Influential Institutions

Most of the institutions that have done research on walking stand out for their importance in various fields worldwide. Table 6 shows a ranking of the 20 institutions with the highest H-index, based on a selection of the 10 journals with the highest number of publications in hiking research. This selection has been made in order to obtain a more accurate assessment of the contributions made by these universities in this field, so that it is also possible to focus attention on those institutions with the strongest presence in hiking. Several universities stand out in these indices, however, Griffith University, Australia, stands out in its influence given by the H-index calculated specifically for this analysis. The rest of the universities have less distance between them. Here, institutions from the United States and Australia, among others, stand out.

### 4.7. Results of the Country Analysis

The production of quality knowledge is a fundamental aspect for the development of countries. In particular, the development of markets and their competitiveness is expected to go hand in hand with advances in knowledge production. This section analyzes the production of knowledge in rambling from the perspective of the countries where it is generated.

Table 7 shows the most influential countries in knowledge production in walking. There is a substantial difference between the first three places, where three English-speaking countries coincide: the United States, Australia and the United Kingdom. These countries have an important reputation in knowledge production, standing out among the countries with the highest scientific production, in terms of quantity and quality.

### 4.8. Results Bibliometric Relationships

The growth in scientific and tourist interest in hiking is a scenario in which the first glimpses of theoretical construction are beginning to be outlined, demanding ever greater rigor for its conceptual development and better understanding. Thus, the research carried out to date constitutes a crucial input for the development of future research.

It is useful to know the structure of academic production in terms of how authors form networks to promote the generation of knowledge based on co-authorship alliances both based on the authors and the countries they represent. It is also of particular interest to consider the conceptual relationships that have developed naturally and implicitly in the academic literature by analyzing co-occurrence based on common keywords considered by the authors. The analysis of these data has been carried out using VOS software version 1.6.8 (Leiden University, Leiden, The Netherlands).

The first analysis was aimed at finding out which authors were linked to others, forming networks. For this purpose, a co-authorship analysis was carried out, since this makes it possible to appreciate the links that materialize with the joint authorship of articles. The unit of analysis was thus taken as the whole number of appearances per author, independent of the number of authors or order of appearance, and authors with a minimum of three articles were considered, without taking into account the number of citations. This resulted in 40 cases, of which 9 contributed to the formation of networks. These networks are shown in the map in Figure 2.

**Figure 3:**
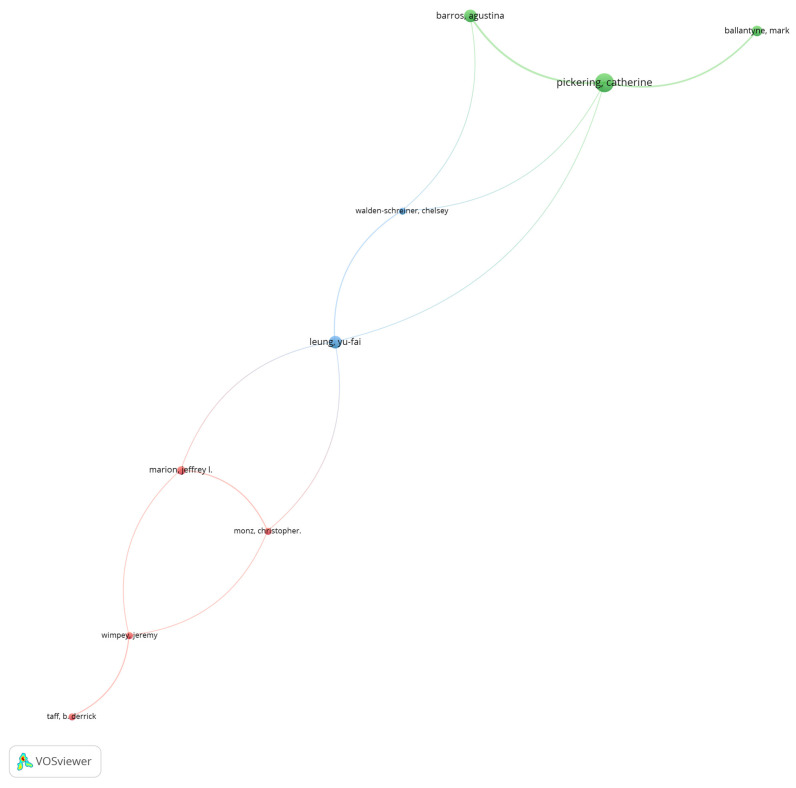
Bibliographic data map of co-authorship analysis (based on authors).

Basically, Figure 3 shows, on the one hand, a larger circle when the author has more co-authors, and on the other hand, the smaller circles show fewer co-authors (note that these relationships are shown to show associations and not the total number of articles written). This map also shows three groups of authors, directly or indirectly related to the work of Professor Pickering of Griffith University, who concentrates the greatest collaboration in this field.

It is interesting to point out that these most productive networks have, in general, authors of different nationalities. This reality supports the belief that international or global networks encourage the development of more and better knowledge, supporting research development policies in emerging countries.

**Figure 4:**
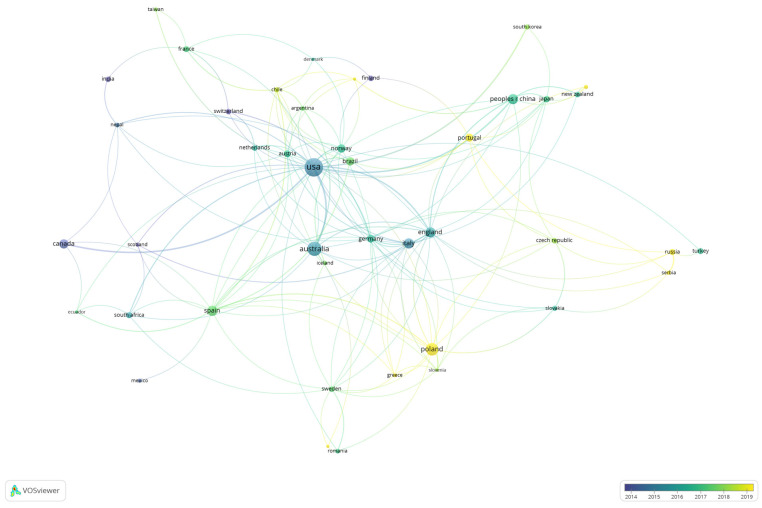
Bibliographic data map of co-authorship analysis (based on countries).

The second analysis in this section sought to delve deeper into the structure of co-authorship, considering the nationalities of the authors. Thus, we again resorted to co-authorship analysis, considering the authors’ countries as the unit of analysis and counting each article independently of the number of authors (or countries) of each article, and without considering the order of appearance of each case. As a minimum, countries with five or more articles were considered in the analysis, resulting in 43 countries with networks. This resulted in the co-authorship map by country shown in Figure 4.

This analysis is consistent with the co-authorship analysis in Figure 4, reaffirming the importance of international networking for the theoretical development of trail tourism. In Figure 4, the evolution of publications by country over time is visually displayed. The size of the circle indicates the quantity of articles published by country. The countries colored in dark blue have articles published around 2014, on the contrary, the countries colored in yellow have articles published around 2019. Thus, countries such as Canada, Switzerland and India can be seen as pioneers in the field of hiking. At the other extreme, we have the countries that have been incorporating this concept in recent decades, such as Poland, Portugal, Russia and Greece among others. Overall it is possible to appreciate through the networks, the links in more traditional countries that have had some participation and influence in the development of more contemporary networks.

The third and final analysis sought to understand the underlying structure of concepts related to hiking as stated by authors in each article, through the choice of keywords. In this way, it is possible to visualize which concepts are related, taking as the main axis each of the research carried out on hiking and the concepts that were considered relevant in each of them.

**Figure 5:**
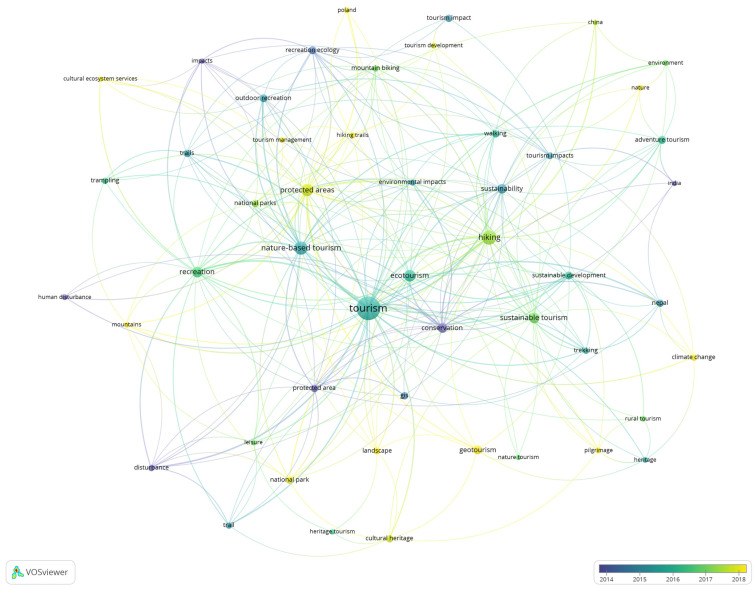
Visualization of networks with common keywords.

To this end, a co-occurrence analysis was carried out using the keywords chosen by the authors as the unit of analysis. In order to favor a visual analysis, we considered those keywords that had a frequency of occurrence of at least seven times, obtaining 50 words that met these criteria. The results of this analysis can be seen in Figure 5.

The result of the keyword analysis is useful to understand the structure of words related to hiking. The size of the circle indicates the quantity of times of appearance by keywords. The keywords colored in dark blue are related with articles published around 2014, on the contrary, the keywords colored in yellow are related with articles published around 2019. In the timeline, it is easy to see that the structure of the research has grown enormously in the last decades. At the more traditional end we see the associated concepts of conservation, protected area, human disturbance, sustainability and recreation ecology, whereas for the more recent concepts we can observe hiking trails, climate change, geotourism, cultural ecosystem services, etc. This also denotes the change in focus around trekking, moving from the need to protect nature (protected area and human disturbance) towards the elements of culture and knowledge of nature (geotourism, climate change and cultural ecosystem services).

It should be noted that these relationships allow us to visualize the concepts that have been most frequently linked to hiking, and by default it also allows us to do the exercise of detecting those concepts that have not captured sufficient attention, but may also play a relevant role in the nomological construction of hiking.

## 5. Discussion

The bibliometric and scientometric analysis of trail tourism presented here establishes the role of the entities in this field (individuals, groups and institutions) with respect to affiliated articles that today show a high growth in knowledge management with quantity, quality and thematic maturity, achieving criticism in the scientific mass [68,81]. The work developed in this article provides an overview of academic research showing part of a universe of knowledge on hiking [8,21,25,82]. A first finding evidences 820 articles published in the main science database WOS, showing an upward trend for hiking in scientific production worldwide from 1991 to 2022.

In general, the results are significant with what one would expect from the development of this activity, which is still in the early stages of its development. In the last 10 years, five Journals stand out with the highest number of citations (*Journal of Environmental Management*, *Journal of Sustainable Tourism* and *Tourism Management Landscape and Urban Planning Tourism Geographies*). These journals demonstrate the division that exists between tourism, environment and sustainability; this does not mean that they are the journals with the most production, but rather that they show their influence in the academy due to the citations they have.

The authors of this research noted as an interesting finding the conceptual context of hiking, advancing in its knowledge from the keywords “hiking”, “trekking” and “trail”, with “tourism”, and motivating the growth of academic research on the subject, pointing to new lines of research such as welfare, health, economic growth in tourism entrepreneurship, ecotourism and nature tourism to name a few, although these advances have been important, there is still room for growth, especially in relation to tourism and wellbeing in a more sustainable way [8,34,39,83]. This is reflected in Figure 5 where the shift in focus around hiking from the need to protect nature to the elements of culture can be seen.

On the other hand, as can be seen in the structure of co-authored research, the number of authors and networks still have much room for development [15,22]. Here it can be hypothesized that with more consolidated networks it would be possible at the same time to bring together scholarly efforts that today are more dispersed, making them more difficult to identify and categorize [10,11,12,13,14,15,16,17,18,19,84,85]. The groups of authors that persist over time are limited, and this is evident from the results in Figure 3 and Figure 4. Figure 3 shows relationships with a minimum of three co-authorships, whereas Figure 4 shows relationships from one co-authorship. This may be limiting with which phenomena associated with hiking are studied. It could also be considered that further development of research from other sources would increase the extrapolation of the results and contribute to consolidate the development of research on hiking.

The results found show that research on walking, although showing a clear upward trend, is still limited. Possibly, this is due to some specific aspects related to the existence of the importance of hiking in terms of tourism and its valuation in terms of well-being [15,33,86]. This would be an important element to explore further.

Meanwhile, the results contribute to broadening the discourse of hiking tourism reinvestigation through the integrated use of classical bibliometric laws and various scientometric techniques [16,69,87], as well as encompassing the production, impact and relationship, laws and techniques commonly used, with a lack of synergy in research on hiking tourism topics. Therefore, it can be argued that trekking, from a perspective of new knowledge and well-being, represents an interesting possibility in terms of knowledge management and leisure-related business (referring to sport, rural, nature and cultural tourism), but it must be managed through correct planning strategies that allow for an increasing professionalization of the sector, avoiding the massive commodification of the resources and spaces in which it is developed. This leads to the search for the establishment of sustainable tourism and development practices that could be the subject of future analyses [88,89,90,91].

## 6. Conclusions

In drawing conclusions, the first point to highlight is the importance of quantitative and qualitative information on trail tourism worldwide. This fact represents a fundamental opportunity when analyzing this field and is undoubtedly one of the great challenges for the future in academic and scientific circles related to trail tourism, opening up incredible perspectives for further research and mitigating the gaps in this context. The relationship between tourism and trails has, in relative terms, a lot of relevant information and should therefore be of residual importance.

The growing consumer demand to “Enjoy tourism” a destination in an authentic way, as well as the increasing popularity of active tourism, means that trail tourism is becoming increasingly important beyond hiking activities, as it shows a destination in its entirety, including local nature and culture. A number of defining characteristics are often indicated in the overall relationship of trail tourism, according to data evidenced by the UNWTO, as shown in the review section of this article trail tourism is now one of the preferred ways of experiencing a destination. It allows tourists to better interact with the local population, nature and culture, generating tranquility and well-being for those who practice it. It also satisfies the growing widespread demand for outdoor activities.

Trail tourism can be developed anywhere as a sustainable tourism offer with a relatively small investment, but with a high economic and social return for both residents and tourist, if properly developed and managed. In this sense, it is observed that trail tourism is beginning to be an important factor in the supply of tourist destinations, knowledge management in universities developing innovation in tourism programmers, health management and the well-being of the people who practice it, and allowing differentiation and greater competitiveness in the international framework. In this sense, major pandemic events are an excellent image campaign for a destination that wants to promote its hiking tourism aspect, since beautiful views, nature and tourism are sources of innovation for the well-being of people in their leisure time.

Additionally, in response to our research purpose the researchers agreed to cross any of these three terms: “hiking”, “trekking” and “trail”, with “tourism”. In this context, it is evident that more and more people are researching and discovering new forms of tourism, which often become popular and mass phenomena, enhancing knowledge management and the management of new forms of wellness tourism. In the particular case of hiking, it can be stated that there is an important connection between leisure and wellbeing. In particular, it is worth pointing out the possibility or convenience of incorporating both facets in the same process in which both private initiative and public management are necessary and clearly complementary, as is the case in most economic activities of a structural nature.

### Limitations

Some limitations must be considered in relation to the methodology used for this study. The information provided, which, although relatively topic-specific, is general within that scope and may have left out elements that could have enriched the analysis much more. However, this work sought to ensure a replicable methodology in order to constitute a comparable contribution over time.

Finally, this work based its analysis on the database provided by WoS, given its importance and scientific acceptance, and was therefore considered a good approximation for the data under study. However, this database is not free of errors, which may have gone unnoticed in this research. Even so, for the development of this work we tried to take measures to validate the data as we progressed with the analyses in order to minimize possible errors.

## Figures and Tables

**Table 1:** Hiking tourism benefits and knowledge management.

Economic	Environmental	Cultural	Social/Health	Authors
Promotes local commerce.	Help raise awareness and protects the environment.	Conservation of cultural heritage and rural traditions.	Promotes enjoying biodiversity in nature.	[17,23,31,32]
It takes advantage of local resources.	Stimulates the cleaning of natural spaces.	Improving the self-esteem of the local population.	Improve health through physical activity.	[6,10,15,33,34,35]
Uses low investment.	Improves knowledge about nature and the environment.		Acts on physical and psychological health.	[36,37,38]
Better income from tourism activity by increasing length of stay at the destination.	Promotes the conservation of conservation.	Promotes duality tourist experience.	Generates a regression to a slower, more human, potentially more relaxed pace of life.	[39,40,41]
Promotes tourism entrepreneurship.			Allows coexistence with others.	[42,43,44,45]
Create new tasks and jobs.			Create an experience of integration with nature.	

Source. Own elaboration based on Kastenholz and Rodrigues, 2007 [39].

**Table 2:** Numbers of citations and ranks of the most-cited journals regarding hiking and trekking (2012–2021).

Source	2012	2013	2014	2015	2016	2017	2018	2019	2020	2021	2012–2021	TC-HT	TP-HT	TC-HT/ TP-HT
Total of Articles Published	27	24	28	34	27	72	92	91	94	124	613	-	783	-
All Journal Citations	523	647	527	734	501	627	856	455	354	121	5345	11,059	-	-
Journal of Environmental Management	0	83	28	156	0	8	0	11	13	0	299	1449	23	63.0
Journal of Sustainable Tourism	83	18	38	0	22	19	56	0	13	3	252	334	15	22.3
Tourism Management	102	15	14	0	69	0	10	15	22	1	248	821	20	41.1
Landscape and Urban Planning	18	0	49	0	0	64	95	0	0	0	226	392	9	43.6
Tourism Geographies	0	0	27	120	51	16	0	0	0	1	215	267	13	20.5
Environmental Monitoring and Assessment	0	0	0	0	82	0	100	8	0	0	190	253	5	50.6
Journal of Outdoor Recreation and Tourism-Research Planning and Management	0	0	0	0	0	52	77	3	42	10	184	184	20	9.2
Tourism Management Perspectives	0	0	0	48	27	65	16	0	0	6	162	162	8	20.3
Annals of Tourism Research	0	0	45	0	0	0	50	3	30	0	128	475	11	43.2
Sustainability	0	0	0	0	8	51	20	20	23	2	124	124	26	4.8
Frontiers in Ecology and the Environment	0	121	0	0	0	0	0	0	0	0	121	121	1	121.0
Geoheritage	11	0	0	0	41	0	22	27	5	1	107	107	13	8.2
Journal of Travel & Tourism Marketing	0	0	0	71	0	19	8	0	0	0	98	98	3	32.7
Plos One	0	0	0	0	89	0	0	0	2	0	91	91	2	45.5
Biological Conservation	0	48	34	0	0	0	0	0	0	0	82	210	5	42.0
Mountain Research and Development	32	0	45	0	0	0	2	1	1	0	81	240	11	21.8
Current Issues in Tourism	0	0	0	52	0	0	0	10	7	11	80	80	7	11.4
Tourist Studies	0	22	34	0	13	0	0	4	0	0	73	73	6	12.2
Scandinavian Journal of Hospitality and Tourism	0	11	0	41	0	0	12	8	0	0	72	134	10	13.4
Environmental Management	0	47	18	0	0	3	0	0	0	0	68	235	7	33.6
Asia Pacific Journal of Tourism Research	0	0	0	0	20	5	37	5	0	0	67	94	7	13.4
Applied Geography	0	0	0	0	0	0	65	0	0	0	65	65	2	32.5
Journal of Heritage Tourism	0	0	0	0	0	52	0	7	3	1	63	63	13	4.8
Journal of Mountain Science	0	0	0	0	0	20	6	19	7	0	52	65	11	5.9
Environments	0	0	0	0	0	0	0	52	0	0	52	52	6	8.7
European Journal of Remote Sensing	0	51	0	0	0	0	0	0	0	0	51	51	1	51.0
Acta Theriologica	0	50	0	0	0	0	0	0	0	0	50	50	1	50.0
Leisure Sciences	49	0	0	0	0	0	0	0	0	0	49	49	1	49.0
Journal of Avian Biology	0	0	0	0	0	47	0	0	0	0	47	47	1	47.0
Annals of the Association of American Geographers	47	0	0	0	0	0	0	0	0	0	47	47	1	47.0
Oryx	0	30	0	14	0	0	0	0	0	0	44	44	3	14.7
Tourism Economics	13	0	0	12	0	4	0	0	7	7	43	68	9	7.6
Wilderness & Environmental Medicine	0	0	33	10	0	0	0	0	0	0	43	43	2	21.5
Biodiversity and Conservation	0	0	18	20	0	0	4	0	0	0	42	135	5	27.0
Ambio	0	0	0	42	0	0	0	0	0	0	42	78	2	39.0

Notes: Abbreviations: TC-HT and TP-HT, total numbers of citations and papers, respectively, in hiking and trekking; and TC/TP, total number of citations by papers in hiking and trekking.

**Table 3:** The most-influential journals regarding hiking and trekking.

R	Name	H-HT	TC-HT	TP-HT	P-HT	≥100	≥50	≥25	≥10	TP	TC	TC/TP	IF	T30	H	R
1	*JEM*	**17**	1449	23	0.15%	5	10	14	19	15.200	62.838	4.13	6.789	3	189	1
2	*TM*	**14**	821	20	0.47%	1	5	8	16	4.238	37.117	8.76	10.967	2	207	2
3	*JST*	**11**	334	15	1.03%	0	1	5	11	1.459	9.404	6.45	7.968	2	90	3
4	*ATR*	**9**	475	11	0.28%	1	4	8	9	3.911	19.981	5.11	9.011	1	192	4
5	*LUP*	**9**	392	9	0.20%	0	3	5	9	4.491	22.745	5.06	6.142	3	169	5
6	*TG*	**9**	267	13	1.54%	0	1	5	9	842	3.605	4.28	6.640	2	58	6
7	*SJHT*	**8**	134	10	2.16%	0	0	1	5	464	1.653	3.56	4.392	0	42	7
8	*G*	**8**	107	13	2.00%	0	0	0	5	649	1.378	2.12	2.680	0	30	8
9	*JORTRPM*	**7**	184	20	7.69%	0	0	2	6	260	818	3.15	2.803	1	18	9
10	*TMP*	**7**	162	8	1.13%	0	0	0	7	709	3.902	5.50	6.586	0	47	10
11	*MRD*	**6**	240	11	0.58%	0	1	4	6	1.900	2.284	1.20	1.194	1	65	11
12	*S*	**6**	124	26	0.06%	0	0	1	2	45.303	71.638	1.58	3.251	1	99	12
13	*EM*	**5**	235	7	0.13%	1	1	1	5	.341	12.193	2.28	3.266	0	131	13
14	*BC*	**5**	210	5	0.05%	0	0	5	5	9.591	39.676	4.14	5.991	1	213	14
15	*APJTR*	**5**	94	7	0.79%	0	0	1	4	888	2.843	3.20	3.677	0	40	15
16	*TE*	**5**	68	9	0.70%	0	0	0	2	1.277	3.682	2.88	4.438	0	42	16
17	*JMS*	**5**	65	11	0.55%	0	0	0	3	2.011	3.315	1.65	2.071	0	39	17
18	*BDC*	**4**	135	5	0.10%	0	1	2	4	5.151	13.966	2.71	3.551	0	135	18
19	*JFNC*	**4**	129	5	0.54%	0	1	2	3	934	2.151	2.30	2.831	0	47	19
20	*JTM*	**4**	118	5	0.20%	0	0	3	4	2.457	5.260	2.14	8.490	0	58	20

It is important to mention the abbreviations: R, ranking; H-HT, h-index only for papers with hiking and trekking, TC-HT and TP-HT, total number of citations in hiking and trekking and total number of papers in hiking and trekking, respectively; % P-HT, percentage of hiking and trekking papers in the journal; TC/TP, total number of citations per paper; ≥100, ≥50, ≥25 and ≥10, number of papers with at least 100, 50, 25 and 10 citations, respectively; TP and TC, total number of papers and total number of citations, respectively; IF, impact factor in 2022; T30, number of papers in the top 30 list in Table 4; H, h-index; JEM, Journal of Environmental Management; TM, Tourism Management; JST, Journal of Sustainable Tourism; ATR, Annals of Tourism Research; LUP, Landscape and Urban Planning; TG, Tourism Geographies; SJHT, Scandinavian Journal of Hospitality and Tourism; G, Geoheritage; JORTRPM, Journal of Outdoor Recreation and Tourism-Research Planning and Management; TMP, Tourism Management Perspectives; MRD, Mountain Research and Development; S, Sustainability; EM, Environmental Management; BC, Biological Conservation; APJTR, Asia Pacific Journal of Tourism Research; TE, Tourism Economics; JMS, Journal of Mountain Science; BDC, Biodiversity and Conservation; JFNC, Journal for Nature Conservation; JTM, Journal of Travel Medicine.

**Table 5:** The 12 most productive and most influential authors in hiking and trekking.

R	Authors	Country	TP-HT	TC-HT	TC/TP	H-HT	H	TP5	TC5	T30	TP	TC
1	Pickering, C	Australia	22	1191	54.14	15	36	5	88	7	127	4595
2	Leung, YF	USA	10	553	55.30	9	20	3	64	2	59	1442
3	Barros, A	Argentina	9	287	31.89	8	12	4	81	4	20	430
4	Wolf, ID	Australia	8	224	28.00	7	14	4	66	1	16	523
5	Croft, DB	Australia	6	180	30.00	5	21	2	22	1	54	1318
6	Ballantyne, M	England	6	174	29.00	6	13	0	0	2	24	402
7	Marion, JL	USA	5	397	79.40	5	21	0	0	0	44	1432
8	Kolodziejczyk, K	Poland	5	14	2.80	2	5	5	14	0	16	52
9	Apollo, M	Poland	5	42	8.40	4	5	5	42	0	16	67
10	Chettri, N	Nepal	4	123	30.75	4	15	1	6	0	45	743
11	Dearden, P	Canada	4	183	45.75	4	27	0	0	0	105	2824
12	Vistad, OI	Norway	4	43	10.75	3	14	2	17	0	27	824

It is important to mention the abbreviations: R, ranking; H-HT, h-index for hiking and trekking only; TC/TP, total number of citations by papers in hiking and trekking; TC-BP and TP-BP, total number of citations in hiking and trekking and total number of papers in hiking and trekking, respectively; TC5 and TP5, total number of citations in hiking and trekking and total number of papers in hiking and trekking in the last 5 years, respectively; T30, number of papers in the Top 30 of Table 3; and TC and TP, total number of citations and total number of papers, respectively. Also notable is the relative difference between the most influential author and those that follow, with the authors’ country of origin standing out, as Australia has three representatives in the top five. This is a sign of a special consideration and interest in hiking in that country’s academic production environment.

**Table 6:** The most influential institutions in hiking and trekking.

University/Institution	Country	H-HT10	TP-HT10	TC-HT10	TC10/TP10	H-HT	TP-HT	TC-HT	TC-HT/ TP-HT
Griffith University	Australia	12	16	765	47.8	15	29	1014	35.0
University of North Carolina	USA	6	8	265	33.1	10	20	583	29.2
North Carolina State University	USA	6	7	260	37.1	10	14	547	39.1
Norwegian Institute Nature Research	Norway	4	6	57	9.5	7	10	145	14.5
University of Georgia	USA	5	5	188	37.6	5	7	196	28.0
University System of Georgia	USA	5	5	188	37.6	5	8	197	24.6
Norwegian University of Life Sciences	Norway	3	4	45	11.3	6	8	129	16.1
United States Department of Agriculture USDA	USA	3	4	48	12.0	3	6	51	8.5
United States Forest Service	USA	3	4	48	12.0	3	6	51	8.5
University of New South Wales Sydney	Australia	4	4	151	37.8	7	9	215	23.9
Consejo Nacional de Investigaciones Cientificas y Tecnicas CONICET	Argentina	3	3	68	22.7	5	8	113	14.1
Mid Sweden University	Sweden	3	3	69	23.0	4	5	90	18.0
University of Innsbruck	Austria	2	3	28	9.3	3	6	54	9.0
University of Nottingham	UK	3	3	61	20.3	3	3	61	20.3
University of Vermont	USA	2	3	45	15.0	4	6	192	32.0
Arizona State University	USA	2	2	29	14.5	2	4	30	7.5
Arizona State University Downtown Phoenix	USA	2	2	29	14.5	2	3	29	9.7

It is important to mention the abbreviations: H-HT10, h-index of hiking and trekking in 10 selected journals; TC-HT and TP-HT; TC/TP, total number of citations of hiking and trekking papers; and H-HT, total number of citations of hiking and trekking, total number of hiking and trekking papers and h-index of hiking and trekking. The ten journals considered are JEM, TM, JST, ATR, LUP, TG, SJHT, G, JORTRPM and TMP.

**Table 7:** The most influential countries in hiking and trekking.

R	Country	TP-HT	H-HT	TC-HT	TC/TP	≥100	≥50	≥25	≥10	TP/i	TC/i	TP-PIB	TC-PIB
1	USA	135	30	2740	20.30	5	18	35	61	4097	83,160	0.006	0.131
2	Australia	82	24	2248	27.41	7	11	24	43	31,923	875,149	0.062	1.693
3	UK	56	17	955	17.05	1	5	12	25	8331	142,081	0.020	0.346
4	Italy	37	15	937	25.32	1	5	12	20	9736	246,545	0.022	0.569
5	Canada	41	15	711	17.34	1	4	10	20	6885	119,387	0.022	0.376
6	Austria	21	12	338	16.10	0	0	5	13	23,550	379,043	0.048	0.780
7	Norway	49	12	616	12.57	1	3	7	16	0.350	4400	0.003	0.042
8	Poland	27	11	348	12.89	1	1	3	12	50,191	646,903	0.075	0.961
9	Switzerland	12	10	291	24.25	0	2	3	10	21,697	526,152	0.045	1.079
10	China	15	10	434	28.93	0	3	7	11	17,367	502,495	0.020	0.577
11	Finland	60	10	337	5.62	0	1	2	11	15,810	88,799	0.101	0.565
12	Spain	42	9	258	6.14	0	1	3	6	8870	54,486	0.033	0.201
13	Germany	12	8	264	22.00	0	1	5	8	6880	151,366	0.013	0.289
14	India	14	8	211	15.07	0	1	4	7	0.100	1507	0.005	0.079
15	Netherlands	24	8	263	10.96	0	1	4	8	2883	31,595	0.006	0.068
16	New Zealand	12	6	134	11.17	0	0	2	5	11,590	129,426	0.022	0.248
17	Sweden	12	6	94	7.83	0	0	0	4	23,602	184,883	0.057	0.446
18	Turkey	15	6	262	17.47	1	2	3	4	1779	31,065	0.021	0.364
19	Japan	9	5	150	16.67	0	2	2	3	3089	51,481	0.267	4.451
20	France	9	5	114	12.67	0	0	2	4	1983	25,123	0.023	0.293
21	Slovakia	12	5	105	8.75	0	1	1	2	21,950	192,061	0.101	0.887
22	South Africa	14	5	136	9.71	0	1	2	3	2361	22,931	0.042	0.405
23	Argentina	14	5	105	7.50	0	1	1	1	2077	15,581	0.005	0.040
24	Nepal	17	5	118	6.94	0	0	1	3	1351	9377	0.003	0.023
25	Portugal	5	4	60	12.00	0	0	0	3	8574	102,891	0.014	0.168
26	Brazil	8	4	98	12.25	0	0	1	3	218,326	2674,490	0.369	4.516
27	Romania	9	4	26	2.89	0	0	0	0	4667	13,481	0.036	0.105
28	Chile	10	4	56	5.60	0	0	0	2	5231	29,295	0.040	0.221
29	Czech Republic	12	4	72	6.00	0	0	1	3	11,216	67,297	0.049	0.294
30	Denmark	25	4	64	2.56	0	0	0	3	24,259	62,102	0.109	0.280

It is important to mention the abbreviations: TP-HT and TC-HT, total numbers of papers and citations, respectively, in hiking and trekking; TC/TP, total number of citations by papers in hiking and trekking; ≥100, ≥50 and ≥25, numbers of papers with at least 100, 50 or 25 citations, respectively; TP/i and TC/i, numbers of papers and citations, respectively, per 10 million inhabitants of the country; and TP/GDP and TC/GDP, ratios of the total numbers of papers and citations, respectively, to GDP in billions of current USD. The GDP and inhabitant data were extracted from The World Bank database.

## Data Availability

Data are available on request from the authors.
